# Effect of dichoptic video game treatment on mild amblyopia – a pilot study

**DOI:** 10.1111/aos.14595

**Published:** 2020-09-30

**Authors:** Peter C. K. Pang, Carly S. Y. Lam, Robert F. Hess, Benjamin Thompson

**Affiliations:** ^1^ School of Optometry The Hong Kong Polytechnic University Kowloon Hong Kong; ^2^ Department of Ophthalmology and Visual Sciences McGill University Montreal Quebec Canada; ^3^ School of Optometry and Vision Science University of Waterloo Waterloo Ontario Canada; ^4^ Centre for Eye and Vision Research (CEVR) Hong Kong

**Keywords:** amblyopia, dichoptic video game, fixation stability, home‐based treatment

## Abstract

**Purpose:**

The effect of contrast‐balanced dichoptic video game training on distance visual acuity (DVA) and stereo acuity has been investigated in severe‐to‐moderate amblyopia, but its effect on mild amblyopia and fixation stability has not been assessed. This pilot study aimed to evaluate the effect of home‐based dichoptic video game on amblyopic eye DVA, stereo acuity and fixation stability in adults with mild amblyopia.

**Methods:**

A randomized single‐masked design was adopted. The active 6‐week home‐based treatment was an anaglyphic, contrast‐balanced dichoptic video game, and the placebo was an identical non‐dichoptic game. Participants (*n* = 23) had mild amblyopia (amblyopic DVA ≤ 0.28 log Minimum Angle of Resolution (logMAR)). The primary outcome was change in amblyopic DVA at 6 weeks postrandomization. Near visual acuity, stereo acuity and fixation stability (bivariate contour eclipse area) were also measured. Follow‐up occurred at 12 and 24 weeks postrandomization.

**Results:**

Mean amblyopic eye DVA was 0.21 ± 0.06 and 0.18 ± 0.06 logMAR for the active (*n* = 12) and placebo (*n* = 11) group, respectively. Amblyopic DVA improved significantly more in the active group (0.09 ± 0.05) than in the placebo group (0.03 ± 0.04 logMAR; p < 0.05). The difference between groups remained at 12 weeks postrandomization (p = 0.04) but not at 24 weeks (p = 0.43). Titmus stereo acuities improved significantly more in the active group (0.40 log arcsec) than in the placebo group (0.09 log arcsec) after 6 weeks of gameplay. The between‐group difference was still present at 24 weeks postrandomization (p = 0.05). There were no differences between groups on any other secondary outcomes.

**Conclusion:**

Home‐based dichoptic video gameplay may be an effective method to improve amblyopic DVA and stereo acuity in mild amblyopia.

## Introduction

Amblyopia is a unilateral or infrequently bilateral neurodevelopmental condition that involves reduced distance visual acuity (DVA) in a healthy eye along with the presence of an amblyogenic factor such as anisometropia, strabismus or stimulus deprivation (Rouse et al. 1994). Amblyopia can be treated using optical correction (Stewart et al. 2004; Pediatric Eye Disease Investigator Group (PEDIG) et al. 2005; PEDIG et al. 2006; Chen et al. 2007; PEDIG et al.^2012^; Gao et al. 2018a); patching or occlusion therapy (PEDIG et al. 2003; Agervi et al. 2013; Tang et al. 2014; Su et al. 2016); and atropine penalization of the nonamblyopic eye (PEDIG et al. 2015). In recent years, a number of additional treatment approaches have been developed including monocular (Levi & Li 2009) and binocular (Hess et al. 2014; Hess & Thompson 2015) perceptual learning.

Binocular perceptual learning approaches, often referred to as binocular or dichoptic treatment, involve splitting visual stimulus elements between the eyes. Amblyopic eye elements are presented at a high contrast and nonamblyopic eye elements at a lower contrast to overcome interocular suppression (Li et al. 2013a) and enable simultaneous perception. Simultaneous perception is crucial because neither eye receives all information necessary to complete the training task. Binocular treatment can be delivered in the form of modified dichoptic video games viewed through lenticular screens on tablet computers (To et al. 2011), virtual reality devices (Knox et al. 2012; Li et al. 2013b; Vedamurthy et al. 2016), shutter glasses (Herbison et al. 2016) or anaglyphic glasses (Hess et al. 2012; Birch et al. 2015; Holmes et al. 2016; Kelly et al. 2016; Gao et al. 2018b).

Improvements in amblyopic eye visual acuity and stereopsis have been reported following binocular treatment in both adults (Hess et al. 2010; Vedamurthy et al. 2015) and children (Birch et al. 2015; Holmes et al. 2016; Kelly et al. 2016) with amblyopia. However, recent clinical trials have reported no effect of binocular treatment in older children and adults with amblyopia (Manh et al. 2018; Gao et al. 2018b; PEDIG et al. 2019). These results may have been due, in part, to difficulties in translating the treatment to the home environment (Holmes 2018; Thompson 2019).

Well‐controlled binocular treatment studies have involved participants with moderate‐to‐severe amblyopia and distance amblyopic eye visual acuity of 0.3 logMAR or worse (Holmes et al. 2016; Kelly et al. 2016; Gao et al. 2018b; PEDIG et al. 2019). However, current amblyopia treatments rarely equate visual acuity between the two eyes in the long term, leaving patients with mild residual amblyopia (Holmes et al. 2007). The effectiveness of binocular treatment in participants with mild amblyopia (interocular DVA difference of at least 0.2 logMAR, but amblyopic eye visual acuity better than 0.3 logMAR) has not been studied.

In addition, outcome measures in binocular treatment studies have primarily focused on visual acuity, stereopsis and interocular suppression (Hess et al. 2010; Hesset al. 2011; To et al. 2011; Hess et al. 2012; Knox et al. 2012; Li et al. 2013a; Birch et al. 2015; Li et al. 2015; Vedamurthy et al. 2015; Manh et al. 2018; Gao et al. 2018b; PEDIG et al. 2019). However, amblyopia has effects that extend beyond visual acuity and binocular vision deficits. One example is impaired fixation stability in the amblyopic eye (Ciuffreda et al. 1979; Gonzalez et al. 2012; Subramanian et al. 2013; Raveendran et al. 2014; Chung et al. 2015; Raveendran et al. 2019). Poorer amblyopic eye fixation stability is associated with poorer visual acuity (Subramanian et al. 2013; Chung et al. 2015) but does not appear to be caused by the visual acuity loss itself (Raveendran et al. 2019). It is unknown whether amblyopic eye fixation stability impairments respond to treatment.

This aim of this pilot study was to assess whether binocular treatment in the form of home‐based dichoptic video gameplay was more effective than a placebo game for improving amblyopic eye DVA in a small group of individuals with mild amblyopia. Secondary outcome measures included near visual acuity (NVA), stereo acuity and fixation stability (bivariate contour ellipse area; BCEA).

## Materials and Methods

### Study design and sample size calculation

Based on an amblyopic eye VA improvement of 0.11 ± 0.08 (effect size = 1.38) after 6 weeks of dichoptic video game treatment (To et al., 2011) and the assumption of no VA change in the placebo group, eight participants per group would provide 90% power to detect a between‐group difference in VA improvement at p < 0.05 with four repeated measurements. Allowing for dropout, 12 participants were recruited in each group.

Block randomization was employed in this study. The first 12 participants were assigned to 6 weeks of home‐based treatment using an active dichoptic video game presented on a handheld iPod touch (Apple, Inc, Cupertino, California, USA) device, and the second 12 were given a placebo video game presented using the same device. Participants were followed up 6 weeks (immediately post‐treatment), 12 and 24 weeks postrandomization. The primary outcome was the change in amblyopic eye DVA at 6 weeks postrandomization.

The study was conducted in parallel with the Binocular Treatment of Amblyopia with Videogames (BRAVO) randomized clinical trial (Gao et al. 2018b) and conformed to the BRAVO study protocol (Guo et al. 2016) with a number of exceptions, some of which related to the smaller scale of this study; there was only one site (The School of Optometry at Hong Kong Polytechnic University), a single‐masked block randomization study design was adopted, fixation stability was assessed as an additional secondary outcome measure, and the visual acuity inclusion criteria were modified to target mild amblyopia.

The study complied with the tenets of the Declaration of Helsinki, and human ethics approvals were obtained from the Hong Kong Polytechnic University Human Ethics Committee. The study was registered at ClinicalTrials.gov (registration number: NCT02995174). Participants and their guardian (if the participants were younger than 18 years of age) provided informed written consent.

### Statistical analysis

An intent‐to‐treat analysis was conducted. The primary outcome of change in amblyopic eye DVA from baseline to immediately post‐treatment was analysed using an ancova with a factor of treatment group (active versus placebo) and covariates of baseline amblyopic eye DVA, age, optical treatment history and patching history. A linear regression model was used to determine the association between the change in amblyopic eye DVA and baseline amblyopic eye DVA, age, training hours, optical treatment history and patching history. The longevity of any statistically significant treatment effects was analysed using the same ancova model conducted separately on the change from baseline at each follow‐up visit. The same analysis approach was applied to each secondary outcome measure. SPSS version 23 (IBM: Armonk, New York, USA) software was used to conduct the analysis.

### Participants

#### Inclusion criteria

1. Aged 7 years or above.
2. Distance visual acuity (DVA) in amblyopic eye equal to or less than 0.28 logMAR, with an interocular difference of at least 0.20 logMAR.
3. Amblyopia could be strabismic, anisometropic or mixed (both strabismic and anisometropic). Strabismic amblyopia was defined as amblyopia in the presence of heterotropia at distance and/or near fixation. Anisometropic amblyopia was defined as amblyopia in the presence of a spherical equivalent (SE) difference ≥0.50 D between the eyes, or a difference of astigmatism in any meridian ≥1.50 D and no strabismus (Guo et al. 2016). Mixed strabismic–anisometropic amblyopia was defined as amblyopia in the presence of both strabismus and anisometropia;
4. Able to complete three successful 30‐second fixation stability measurements with a MP‐1 micro‐perimeter for each eye.
5. Able to align the nonius cross (≤10 mm horizontal error and 5 mm vertical error) in the iPod game (the dichoptic game images could only be displaced by a limited amount to facilitate binocular alignment due to the small iPod screen size (Guo et al. 2016); and
6. Stable visual acuity with full refractive correction (Guo et al. 2016).

#### Exclusion criteria

1. Myopia of SE power equal to or more negative than −6.00 D in either eye.
2. Previous intraocular surgery.
3. Ocular pathology (e.g. media opacities or retinal lesions) other than strabismus.
4. Previous or current history of a neurological problem.

Baseline and outcome assessments were performed by a registered optometrist (author PP). If a participant required a change to their habitual refractive correction to meet study refractive correction criteria (Guo et al. 2016), a new correction was provided and participants were followed up at 4‐week intervals until amblyopic eye DVA was stable (<0.10 logMAR change between visits). The baseline assessment was conducted once DVA was stable.

Patching history was estimated using self‐reported information from participants. Optical correction history was the number of years that the participant had been wearing optical correction prior to the baseline assessment.

Distance visual acuity (DVA) was measured using an Electronic Early Treatment Diabetic Retinopathy Study (ETDRS) test delivered on an Electronic Visual Acuity Tester system (Jaeb Center for Health Research) at 3 m (Beck et al. 2003; Cotter et al. 2003). Near visual acuity (NVA) was measured at 40 cm using the Lighthouse NVA Chart. All visual acuities were recorded using the logMAR format. Stereo acuity was measured using the Titmus stereo test and the Randot Preschool stereo test administered in accordance with the manuals. Fixation stability was measured using a Nidek MP‐1 micro‐perimeter (Nidek Co. Ltd., Gamagori, Aichi, Japan). Participants were asked to fixate monocularly on a red circular ring (1°) for 30 seconds. Fixation stability was calculated by determining a contour ellipse that circumscribed 95% of the area containing all fixation points (Subramanian et al. 2013). In order to minimize the intrapersonal variance, three fixation stability measurements were made for each eye and the best record (smallest value) for each eye was used for analysis. Eligible participants were randomized following their baseline assessment.

### Intervention

The active dichoptic video game was a modified version of Tetris played on an iPod touch device (To et al. 2011; Gao et al. 2018b). Participants played the game wearing red/green glasses. All participants completed training on how to operate and play the game. At the start of each treatment session, participants aligned a nonius cross on the iPod screen and then commenced gameplay. The amblyopic eye saw the falling blocks and the fellow eye saw the static blocks that tessellated with the falling blocks. Lower rows of static blocks were presented to both eyes. Treatment began with fellow eye blocks presented at 20% contrast and amblyopic eye blocks at 100% contrast. Fellow eye contrast was increased each day following an automatic algorithm based on successful gameplay, and the game software recorded the contrast changes and duration of gameplay. Participants were instructed to play the game for 1 hr every day for 6 weeks. The training could be split across several sessions in the same day. The iPod was returned at the 6‐week postrandomization visit.

The placebo game was identical to the active game except that it was not dichoptic (both eyes saw all game blocks at 100% contrast). Participants received the same instructions as the active group and wore red/green glasses during gameplay. Nonius cross alignment was the same in both the active and placebo game versions.

**Fig. 1:**
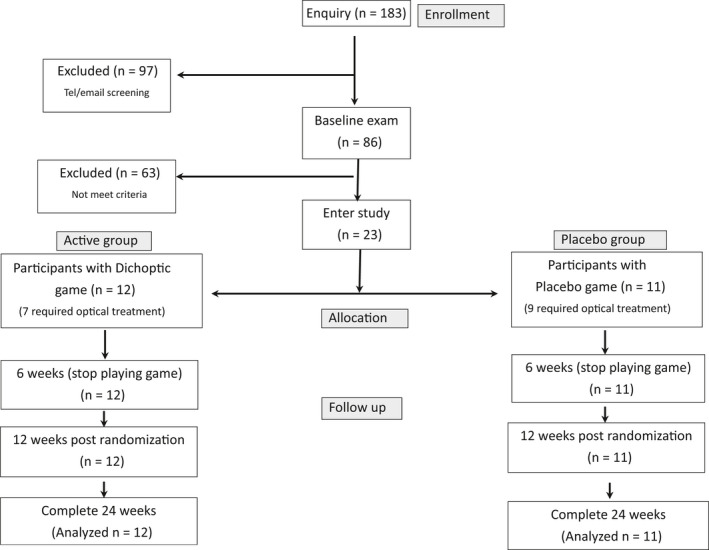
Consort flow chart of this study.

Figure 1 shows the CONSORT flow chart.

## Results

### DVA changes after optical treatment

Sixteen participants from both groups (seven in active and nine in placebo) were required to wear new prescription glasses. Distance visual acuity (DVA) for all participants was stable (visual acuity change was <0.10 log MAR) at the 4‐week follow‐up visit except for one participant (P4) who stabilized after 8 weeks of wearing optical correction. Distance visual acuity (DVA) improved significantly by 0.04 ± 0.05 logMAR in the amblyopic eye after optical treatment (*t*_15_ = 3.07, p < 0.05), while there was no significant change in the fellow eye (0.01 ± 0.05 log MAR; *t*_15_ = 0.56, p = 0.59).

Twenty‐three participants were randomized to the active (*n* = 12) or placebo (*n* = 11) group. All participants could align the nonius cross on the iPod screen. There were no statistically significant differences in baseline age, patching history, optical correction history, DVA, NVA, Titmus stereo acuity, Randot stereo acuity or BCEA between the two groups (all p > 0.05). The baseline characteristics and the DVA of each participant are provided in Table 1.

**Table 1:** Baseline characteristics and distance visual acuity (DVA) of each participant.

Participant A = active P = placebo	Age (years)	Gender (M/F)	Amblyopic eye (R/L)	Patching history (hr)	Optical correction history (years)	Amblyopia type	Given new optical correction	Amblyopic eye DVA before optical correction (log MAR)	Amblyopic eye DVA at randomization (log MAR)	Fellow eye DVA before optical correction (log MAR)	Fellow eye DVA at randomization (log MAR)
A1	22	M	R	13 140	19	Mixed	N	n/a	0.14	n/a	−0.22
A2	46	M	L	0	38	Aniso	Y	0.16	0.20	−0.22	−0.28
A3	41	F	R	0	13	Aniso	Y	0.28	0.24	−0.06	−0.06
A4	40	F	L	0	6	Aniso	Y	0.20	0.18	−0.04	−0.06
A5	30	M	L	1440	24	Aniso	Y	0.08	0.10	−0.20	−0.16
A6	51	M	R	0	35	Aniso	N	n/a	0.22	n/a	−0.30
A7	9	F	R	90	0.5	Aniso	N	n/a	0.22	n/a	0.02
A8	8	M	R	180	0.5	Aniso	N	n/a	0.26	n/a	−0.02
A9	26	M	R	1080	22	Aniso	Y	0.22	0.14	−0.12	−0.10
A10	10	F	L	720	0	Aniso	Y	0.28	0.28	−0.12	−0.12
A11	13	M	R	15 552	11	Mixed	Y	0.34	0.28	−0.12	−0.10
A12	11	M	L	720	4	Strabismic	N	n/a	0.24	n/a	0.00
Mean (SD)	26 (16)	8M/4F	7R/5L	2744 (5465)	14 (13)	9 aniso 1 strabismic 2 mixed	7/12 (58%)		0.21 (0.06)		−0.12 (0.11)
P1	18	F	L	1440	12	Aniso	Y	0.14	0.16	−0.16	−0.14
P2	27	M	L	360	23	Aniso	Y	0.16	0.12	−0.12	−0.16
P3	29	F	L	360	21	Aniso	Y	0.28	0.24	−0.08	0.02
P4	9	F	R	1080	2	Aniso	Y	0.32, after 4 weeks: 0.16	After 8 weeks: 0.16	−0.08, after 4 weeks: −0.10	After 8 weeks: −0.10
P5	37	M	R	0	21	Aniso	Y	0.12	0.06	−0.22	−0.28
P6	24	M	L	2160	20	Aniso	Y	0.24	0.24	−0.02	0.00
P7	31	F	L	720	25	Aniso	Y	0.20	0.16	−0.04	−0.04
P8	14	M	L	14 400	11	Mixed	N	n/a	0.18	n/a	−0.14
P9	43	F	L	720	0	Aniso	N	n/a	0.26	n/a	−0.02
P10	27	M	L	360	23	Strabismic	Y	0.28	0.22	0.04	0.00
P11	31	M	L	360	21	Aniso	Y	0.26	0.18	−0.06	−0.14
Mean (SD)	26 (10)	6M/5F	2R/9L	1996 (4159)	16 (9)	9 aniso 1 strabismic 1 mixed	9/11 (82%)		0.18 (0.06)		−0.09 (0.09)

### Treatment adherence

The mean hours of gameplay within the 6‐week treatment period were 37 ± 10 and 33 ± 10 hr for the active and placebo groups, respectively. There was no significant difference (*t*_10_ = 0.72, p = 0.5) in treatment hours between the two groups; 10 out of 12 (83%) participants in the active group and 6 out of 11 (55%) in the placebo group reached 75% of the prescribed hours.

### DVA changes after video game treatment (primary outcome)

After accounting for baseline amblyopic eye DVA, age, optical treatment history and patching history within the ancova model, the adjusted mean change in amblyopic eye DVA from baseline to immediately after treatment was 0.09 log MAR (95% CI: 0.07–0.12) for the active group and 0.03 logMAR (95% CI: 0.00–0.06) for the placebo group. The difference between groups was statistically significant (*F*_1,17_ = 11.95, p = 0.003, *η*p^2^ = 0.41). Linear regression revealed no statistically significant association between improvement in amblyopic eye DVA and number of training hours (*B* = 0.00, p = 0.93), baseline amblyopic eye DVA (*B* = 0.01, p = 0.98), age (*B* = 0.00, p = 0.58), optical treatment history (*B* = 0.00, p = 0.45) or patching history (*B* = 0.00, p = 0.69) in the active group.

**Fig. 2:**
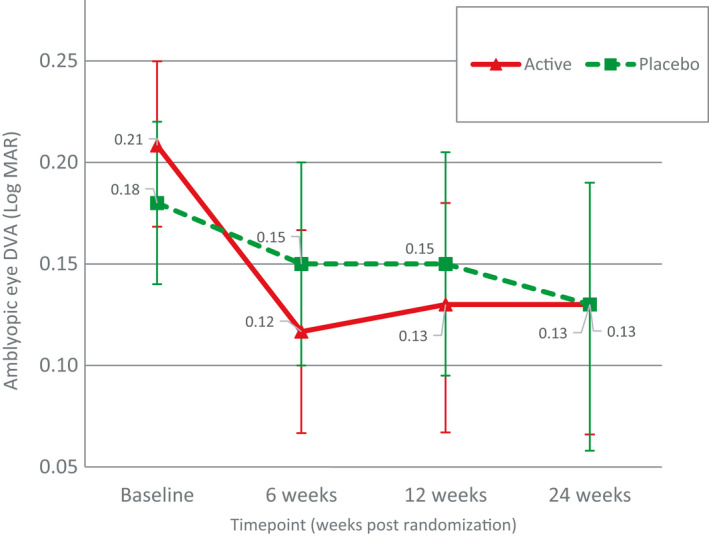
Amblyopic eye distance visual acuity (DVA) from the active and placebo groups at four visits (error bars indicate the upper and lower bounds for means).

The change in amblyopic eye DVA from baseline remained significantly different between the two groups at the 12‐week postrandomization visit (adjusted mean improvement: active group: 0.09 logMAR, 95% CI: 0.05 to 0.12; placebo group: 0.03 logMAR, 95% CI: −0.01 to 0.07; *F*_1,17_ = 5.17, p = 0.04, *η*p^2^ = 0.23). The difference between groups was not significant at the 24‐week postrandomization visit (adjusted mean improvement: active group: 0.08 logMAR, 95% CI: 0.03–0.12; placebo: 0.05 logMAR, 95% CI: 0.01–0.10; *F*_1,17_ = 0.65, p = 0.43, *η*p^2^ = 0.04). Figure 2 shows the amblyopic eye DVA in the two groups at the four visits.

### Secondary outcomes after video game treatment

#### Near visual acuity

The change in amblyopic eye NVA immediately after treatment did not differ significantly between groups (adjusted mean improvement: active group: 0.08 logMAR, 95% CI: 0.04–0.12; placebo group: 0.05 logMAR, 95% CI: 0.01 to 0.09; *F_1_*_,17_ = 0.99, p = 0.33, *η*p^2^ = 0.06).

#### Fixation stability

The change in amblyopic eye BCEA immediately after treatment also did not differ significantly between the two groups (adjusted mean improvement: active group: −0.06 log deg^2^, 95% CI: −0.18 to 0.06; placebo group: −0.02 log deg^2^, 95% CI: −0.14 to 0.10; *F*_1,16_ = 0.24, p = 0.63, *η*p^2^ = 0.01).

#### Stereo acuity

**Fig. 3:**
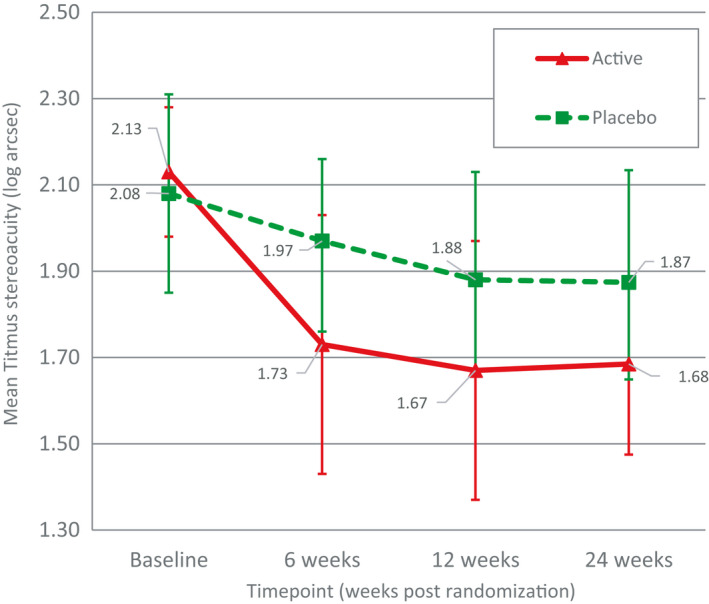
Titmus stereo acuity from the active and placebo groups at four visits (error bars indicate the upper and lower bounds for means).

There was a significant difference between groups in the change in Titmus stereo acuity immediately after treatment (adjusted mean improvement: active group: 0.40 log arcsec, 95% CI: 0.19–0.62; placebo group: 0.09 log arcsec, 95% CI: −0.13 to 0.32; *F*_1,16_ = 4.39, p = 0.05, *η*p^2^ = 0.22). Linear regression revealed no significant association between the change in Titmus stereo acuity and hours of gameplay (*B* = 0.01, p = 0.52), baseline Titmus stereo acuity (*B* = −0.93, p = 0.09), optical treatment history (*B* = 0.03, p = 0.07) or patching history (*B* = 0.00, p = 0.07) for the active group. There was a significant association between change in Titmus stereo acuity and age (*B* = −0.03, p = 0.03), whereby younger participants exhibited a greater improvement. The change in stereopsis from baseline remained significantly different between groups at the 12‐week postrandomization visit (adjusted mean improvement: active group: 0.48 log arcsec, 95% CI: 0.29–0.66; placebo group: 0.17 log arcsec, 95% CI: −0.02 to 0.37; *F*_1,16_ = 5.43, p = 0.03, *η*p^2^ = 0.25) as well as at the 24‐week postrandomization visit (adjusted mean improvement: active group: 0.46 log arcsec, 95% CI: 0.27–0.65; placebo group: 0.18 log arcsec, 95% CI: −0.01 to 0.38; *F*_1,16_ = 4.42, p = 0.05, *η*p^2^ = 0.22). These results indicated a significant improvement in Titmus stereo acuity that lasted 18 weeks after cessation of active treatment. Figure 3 shows the Titmus stereo acuity of the participants in the two groups at the four visits.

There was also significant difference between groups in Randot Preschool Stereo Acuity change immediately after treatment (active group: 0.23 log arcsec, 95% CI: 0.00–0.46; placebo group: −0.10 log arcsec, 95% CI: −0.34 to 0.14; *F*_1,16_ = 4.42, p = 0.05, *η*p^2^ = 0.22). However, the treatment effect was not present at the week 12 (active group: 0.10 log arcsec, 95% CI: −0.12 to 0.31; placebo group: −0.04 log arcsec, 95% CI: −0.26 to 0.18; *F*_1,16_ = 0.80, p = 0.38, *η*p^2^ = 0.05) and the week 24 visits (active group: 0.10 log arcsec, 95% CI: −0.28 to 0.47; placebo group: −0.30 log arcsec, 95% CI: −0.70 to 0.10; *F*_1,16_ = 2.23, p = 0.16, *η*p^2^ = 0.12).

#### Fellow eye

There were no significant between‐group differences immediately after treatment for change in fellow eye DVA (adjusted mean improvement: active group: 0.01 logMAR, 95% CI: −0.02 to 0.04; placebo group: 0.02 logMAR, 95% CI: −0.01 to 0.05; *F*_1,16_ = 0.18, p = 0.68, *η*p^2^ = 0.01), fellow eye NVA (adjusted mean improvement: active group: 0.02 logMAR, 95% CI: −0.01 to 0.05; placebo group: 0.01 logMAR, 95% CI: −0.02 to 0.04; *F*_1,16_ = 0.41, p = 0.53, *η*p^2^ = 0.02) or fellow eye BCEA (adjusted mean improvement: active group: −0.06, 95% CI: −0.19 to 0.08; placebo group: −0.05, 95% CI: −0.20 to 0.09; *F*_1,16_ = 0.00, p = 0.99, *η*p^2^ = 0.00).

The outcome measurements for each participant at each different visit are provided in Table 2.

**Table 2:** Outcome measurements of each participant.

Participant A = active P = placebo	Training hours	Amblyopic eye DVA (log MAR)				Amblyopic eye NVA (log MAR)				Titmus stereo acuity (log arcsec)				Randot preschool (log arcsec)				Amblyopic eye BCEA (log deg^2^)			
		Week 0	Week 6	Week 12	Week 24	Week 0	Week 6	Week 12	Week 24	Week 0	Week 6	Week 12	Week 24	Week 0	Week 6	Week 12	Week 24	Week 0	Week 6	Week 12	Week 24
A1	25	0.14	0.10	0.08	0.10	0.36	0.20	0.14	0.18	2.20	2.20	1.80	1.80	4.00	4.00	4.00	4.00	−0.37	−0.42	−0.47	−0.54
A2	43	0.20	0.12	0.16	0.04	0.42	0.36	0.40	0.42	2.20	1.30	1.30	1.51	2.60	2.00	2.60	2.30	0.11	0.10	0.35	0.10
A3	36	0.24	0.22	0.16	0.20	0.66	0.44	0.72	0.52	2.30	2.60	2.20	2.20	4.00	2.90	4.00	4.00	0.03	0.33	0.11	0.06
A4	40	0.18	0.08	0.10	0.12	0.32	0.32	0.38	0.38	1.80	1.51	1.30	1.40	2.00	2.00	2.00	2.00	0.00	−0.21	−0.18	−0.17
A5	13	0.10	−0.04	0.00	−0.02	0.20	0.16	0.10	0.12	2.00	1.60	1.40	1.40	2.00	2.00	2.00	1.60	−0.55	−0.70	−0.64	−0.28
A6	48	0.22	0.10	0.08	0.10	0.72	0.60	0.30	0.30	2.00	1.80	1.40	1.60	2.60	2.60	2.30	2.30	−0.05	0.10	0.19	0.02
A7	32	0.22	0.16	0.22	0.26	0.26	0.22	0.32	0.38	2.30	1.60	1.60	1.40	2.30	1.78	1.78	1.78	−0.15	−0.03	−0.17	−0.04
A8	43	0.26	0.08	0.06	0.10	0.22	0.12	0.12	0.22	2.00	1.40	1.30	1.40	2.00	1.78	1.60	2.00	0.50	0.58	0.51	0.19
A9	48	0.14	0.02	0.00	0.04	0.12	0.04	0.10	0.14	2.20	1.51	1.60	1.60	2.90	2.90	2.90	4.00	−0.60	−0.40	−0.68	−0.80
A10	41	0.28	0.24	0.28	0.30	0.44	0.34	0.40	0.44	2.20	1.30	2.20	2.20	4.00	4.00	4.00	4.00	−0.13	0.14	−0.08	−0.09
A11	36	0.28	0.14	0.18	0.18	0.40	0.26	0.30	0.30	1.70	1.30	1.30	1.51	4.00	4.00	4.00	4.00	0.02	−0.33	−0.14	−0.15
A12	42	0.24	0.18	0.20	0.18	0.50	0.46	0.52	0.54	2.60	2.60	2.60	2.20	4.00	4.00	4.00	4.00	0.40	0.54	0.93	−0.04
Mean (SD)	37 (10)	0.21 (0.06)	0.12 (0.08)	0.13 (0.09)	0.13 (0.09)	0.39 (0.18)	0.29 (0.16)	0.32 (0.19)	0.33 (0.14)	2.13 (0.24)	1.73 (0.48)	1.67 (0.44)	1.68 (0.33)	3.03 (0.89)	2.83 (0.94)	2.93 (1.00)	3.00 (1.06)	−0.07 (0.33)	−0.02 (0.40)	−0.02 (0.47)	−0.14 (0.28)
P1	44	0.16	0.16	0.16	0.18	0.38	0.20	0.20	0.22	2.20	2.20	1.70	2.20	2.90	4.00	2.90	4.00	−0.27	−0.44	−0.16	−0.16
P2	43	0.12	0.06	0.06	−0.02	0.10	0.12	0.16	0.12	2.20	2.20	2.20	2.00	4.00	4.00	4.00	2.60	−0.27	−0.31	−0.21	−0.17
P3	30	0.24	0.16	0.16	0.16	0.30	0.18	0.22	0.22	2.00	1.80	1.40	1.30	2.60	2.30	2.00	2.60	−0.17	0.00	−0.14	−0.10
P4	26	0.16	0.12	0.22	0.12	0.16	0.16	0.14	0.18	1.60	1.70	1.70	2.00	4.00	4.00	4.00	4.00	0.13	0.34	0.19	0.15
P5	48	0.06	0.04	−0.08	0.04	0.32	0.28	0.22	0.24	1.30	1.40	1.40	1.30	1.60	1.60	1.60	2.00	−0.41	−0.33	−0.21	−0.25
P6	30	0.24	0.22	0.24	0.26	0.36	0.36	0.32	0.36	2.60	2.20	2.20	2.20	2.90	2.90	4.00	4.00	−0.04	−0.32	−0.07	−0.15
P7	18	0.16	0.10	0.12	0.06	0.26	0.18	0.32	0.20	2.20	1.60	1.60	1.40	4.00	4.00	4.00	4.00	−0.96	−0.66	−0.33	−0.55
P8	22	0.18	0.10	0.20	−0.04	0.36	0.28	0.26	0.24	2.20	2.20	2.20	2.20	4.00	4.00	4.00	4.00	0.11	−0.24	−0.01	0.13
P9	42	0.26	0.32	0.24	0.26	0.38	0.42	0.32	0.44	2.20	2.00	2.00	1.80	2.90	2.60	2.90	4.00	−0.46	−0.11	0.00	−0.34
P10	38	0.22	0.20	0.16	0.18	0.26	0.24	0.24	0.30	2.30	2.20	2.30	2.20	4.00	4.00	4.00	4.00	−0.59	−0.54	0.01	−0.14
P11	24	0.18	0.18	0.16	0.18	0.14	0.22	0.20	0.34	2.00	2.20	2.00	2.00	2.30	2.60	2.30	2.60	0.03	0.20	0.13	0.44
Mean (SD)	33 (10)	0.18 (0.06)	0.15 (0.08)	0.15 (0.09)	0.13 (0.10)	0.27 (0.10)	0.24 (0.09)	0.24 (0.06)	0.26 (0.09)	2.08 (0.35)	1.97 (0.30)	1.88 (0.34)	1.87 (0.37)	3.20 (0.85)	3.27 (0.89)	3.25 (0.94)	3.44 (0.80)	−0.26 (0.33)	−0.22 (0.30)	−0.07 (0.16)	−0.10 (0.27)

BCEA = bivariate contour ellipse area; DVA = distance visual acuity, NVA = near visual acuity.

#### Change in interocular contrast

**Fig. 4:**
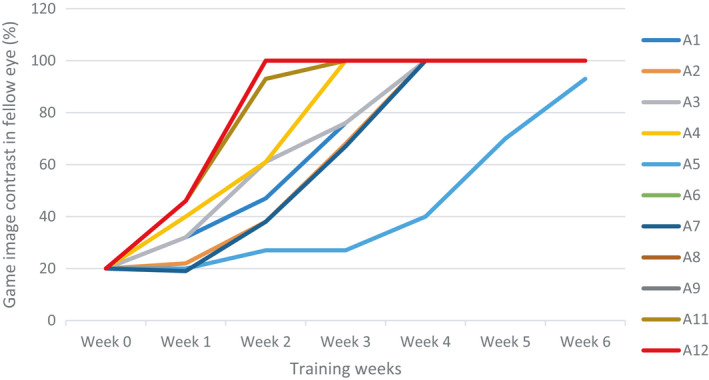
Change of image contrast in the iPod game in the fellow eye in the active group.

In the active group, 10 out of the 12 participants reached 100% fellow eye contrast at the end of treatment, and one participant achieved 93% (Fig. 4). One participant's iPod log file (A10) was not stored due to a technical error during weeks 4–6, and we treated this as missing data. The contrast of the images between the two eyes was equal throughout the whole training period in the placebo group, and therefore, change in fellow eye contrast was irrelevant.

#### Adverse effects

No diplopia or discomfort was reported. Two participants reported mild adaptation difficulty during the initial phase of optical treatment.

## Discussion

It is worth asking the question, ‘why treat mild cases of amblyopia? Firstly, they are particularly difficult to treat (Tsirlin et al. 2015) and secondly, what real world benefits does an improvement from 20/40 to 20/20 impart to a person with amblyopia who has 20/20 visual acuity in the other eye?’ The amblyopic eye is suppressed regardless of its acuity, and to restore binocular function, fusion has to be able to compete with and overcome interocular suppression. This is the rationale of a binocular or dichoptic treatment (Hess & Thompson 2015). So, the answer to the question posed above is that the one strong reason to treat mild amblyopia is to restore binocular vision, at least fusional abilities and hopefully 3D abilities, because this results in better fine motor skills (Webber et al. 2016). This provides real‐world advantages that individuals with amblyopia lack. In concordance with previous studies of binocular therapy (To et al. 2011; Li et al. 2013b; Hess et al. 2014), in this study of mild amblyopia, binocular single vision was present in 90% of participants as reflected in their ability to play the dichoptically presented video game at equal high contrast in both eyes by the end of treatment (Fig. 4). This suggests that fusion has overcome cortical suppression and that the dichoptic contrast balancing strategy was successful. No cases of resistant suppression or diplopia were seen. The sustained improvement (18 weeks post‐treatment) in the Titmus stereo acuity in this study is a further evidence for the binocularity enhancement. In a group where improvements in amblyopic eye visual acuity are necessarily small, hard to achieve and of questionable relevance, the restoration of binocular vision is an important and significant achievement.

We observed a significant improvement in amblyopic eye DVA and stereo acuity (both Titmus and Randot stereo acuities) following active binocular treatment compared to a placebo treatment in our mild amblyopia participants. As this is the first study reporting the treatment effect of binocular treatment in mild amblyopia, we cannot compare our results directly with prior studies, all of which have focused on moderate‐to‐severe amblyopia (To et al. 2011; Hess et al. 2012; Birch et al. 2015; Vedamurthy et al. 2015; Holmes et al. 2016; Kelly et al. 2016; Manh et al. 2018; Gao et al. 2018b; PEDIG et al. 2019). That being said, it is notable that the treatment adherence we observed (83% of active group participants reached 75% of the prescribed treatment hours) is substantially higher than many previous binocular treatment studies, including those that found no treatment effect in moderate‐to‐severe amblyopia (Manh et al. 2018; Gao et al. 2018b; PEDIG et al.^2019^). However, we did not observe a dose–response relationship for the active group. This may have been due to our small sample size, and the treatment hours in our participants were skewed towards good adherence. In addition, it is currently impossible to precisely define treatment dose for binocular treatment in the home environment because the frequency and duration of gazes away from the treatment device screen are not recorded. The distribution of gameplay throughout the day (one long session versus many short sessions) is also not accounted for. Therefore, duration of gameplay as recorded by the treatment device may be only a crude estimate of dose. The development or more sophisticated adherence monitoring systems or supervised in‐office studies are required to directly address the issue of dose–response for binocular treatment.

Although there was not a statistically significant between‐group difference in absolute number of hours played, a smaller percentage of participants in the placebo group (55%) than the active group (83%) completed 75% of the prescribed dose (31.5 hr completed from 42 hr prescribed). As shown in Table 2, two participants from the placebo group failed to meet the 75% threshold by only 1.5 hr. If these participants had played an extra 1.5 hr, the placebo group percentage of participants who completed 75% of the prescribed treatment dose would have increased to 72%, a closer match to the active group. In a much larger sample of participants with moderate‐to‐severe amblyopia, Gao and colleagues found no significant differences in adherence between active and placebo binocular treatment groups (Gao et al. 2018b).

We found that the significant between‐treatment‐group difference in amblyopic eye DVA was no longer present at the 24‐week postrandomization follow‐up. With reference to Fig. 2, we speculate that this effect was not due to a regression effect in the active group, but rather a gradual improvement in the placebo group. There were more participants who required new optical prescription in the placebo (82%, nine out of 11) than the active (58%, seven out of 12) group before the randomization. Hence, it is possible that the placebo group exhibited a greater, gradual optical treatment effect (Stewart et al. 2004; PEDIG et al. 2005; PEDIG et al. 2006; PEDIG et al. 2012; Gao et al. 2018a) than the active group that was not controlled for by our stability criteria of <0.10 logMAR change over 4 weeks.

### Fixation stability

We did not find any effect of binocular treatment on fixation stability. However, our sample of participants with mild amblyopia did not exhibit reduced amblyopic eye fixation stability (BCEA values did not differ between the fellow and amblyopic eyes) at the baseline. This observation (reasonable fixation stability in mild amblyopic eye) is in general agreement with previous reports, indicating that poorer fixation stability is correlated with poorer visual acuity level in amblyopia (Subramanian et al. 2013; Raveendran et al. 2019). Our visual acuity inclusion criteria may not have allowed us to capture individuals with fixation stability impairments.

### Stereo acuity

We observed a significant improvement in stereo acuity measured by the two stereo tests immediately after the 6‐week binocular treatment in the active group. However, only the improvement on the Titmus stereo test lasted up to 18 weeks post‐treatment. The Titmus stereo test measures local stereopsis, whereas the Randot preschool stereo test measure global stereopsis (Fawcett & Birch 2003). In addition, the Titmus stereo test has potential monocular cues whereas the Randot test does not. These differences between the tests may underlie the different treatment outcomes.

### Limitation of this study

The sample size for this pilot study was much smaller than that of recent randomized clinical trials. In addition, our results suggest that stricter DVA stability criteria and a longer optical treatment period are required for participants with mild amblyopia as visual acuity improvements may be particularly gradual in this group. However, despite these limitations, we did observe positive treatment effects within a placebo‐controlled design. Our results stand alone and may also serve to power a more substantial randomized controlled trial study designed to test treatments for mild amblyopia. An additional limitation is that the clinician conducting the outcome measures was not masked to participants' treatment group allocation. This was due to resource limitations and introduced a source of bias.

## Conclusion

Home‐based dichoptic video gameplay may be an effective method to improve amblyopic eye DVA and stereo acuity in mild amblyopia.
